# Transcriptome Analysis Reveals Multiple Genes and Complex Hormonal-Mediated Interactions with PEG during Adventitious Root Formation in Apple

**DOI:** 10.3390/ijms23020976

**Published:** 2022-01-17

**Authors:** Shaohuan Li, Muhammad Mobeen Tahir, Tong Wu, Lingling Xie, Xiaoyun Zhang, Jiangping Mao, Anam Ayyoub, Libo Xing, Dong Zhang, Yun Shao

**Affiliations:** 1 Yangling Sub-Center of National Center for Apple Improvement, College of Horticulture, Northwest Agriculture & Forestry University, Yangling, Xianyang 712100, China; lishaohuan@nwafu.edu.cn (S.L.); mubeentahir924@gmail.com (M.M.T.); wutong@nwafu.edu.cn (T.W.); 2019055163@nwafu.edu.cn (L.X.); mjp588@163.com (J.M.); Libo_xing@nwafu.edu.cn (L.X.); 2 The Key Laboratory of Special Fruits and Vegetables Cultivation Physiology and Germplasm Resources Utilization in Xinjiang Production and Construction Group, College of Agriculture, Shihezi University, Shihezi 832003, China; zh_xiaoyun@sina.cn; 3 College of Life Sciences, Northwest Agriculture & Forestry University, Yangling, Xianyang 712100, China; anamayyoub@nwsuaf.edu.cn

**Keywords:** apple rootstock, AR formation, PEG, stress, hormones, mRNA analysis

## Abstract

Adventitious root (AR) formation is a bottleneck for the mass propagation of apple rootstocks, and water stress severely restricts it. Different hormones and sugar signaling pathways in apple clones determine AR formation under water stress, but these are not entirely understood. To identify them, GL-3 stem cuttings were cultured on polyethylene glycol (PEG) treatment. The AR formation was dramatically decreased compared with the PEG-free control (CK) cuttings by increasing the endogenous contents of abscisic acid (ABA), zeatin riboside (ZR), and methyl jasmonate (JA-me) and reducing the indole-3-acetic acid (IAA) and gibberellic acid 3 (GA3) contents. We performed a transcriptomic analysis to identify the responses behind the phenotype. A total of 3204 differentially expressed genes (DEGs) were identified between CK and PEG, with 1702 upregulated and 1502 downregulated genes. Investigation revealed that approximately 312 DEGs were strongly enriched in hormone signaling, sugar metabolism, root development, and cell cycle-related pathways. Thus, they were selected for their possible involvement in adventitious rooting. However, the higher accumulation of ABA, ZR, and JA-me contents and the upregulation of their related genes, as well as the downregulation of sugar metabolism-related genes, lead to the inhibition of ARs. These results indicate that AR formation is a complicated biological process chiefly influenced by multiple hormonal signaling pathways and sugar metabolism. This is the first study to demonstrate how PEG inhibits AR formation in apple plants.

## 1. Introduction

Apple (*Malus domestica*) is a broadly cultivated profitable fruit tree. High-density plantation techniques are needed, which largely depend on the use of dwarf rootstocks. In the Loess Plateau region of China, numerous apple orchards have poor irrigation systems or no irrigation systems at all. To achieve dwarf cultivation, some orchards were constructed with interstocks (dwarfed intermediate anvils). Usually, the length of interstocks is 25 cm. The depth of interstocks to be buried in the soil is based on soil fertility status. After interstocks are planted in the ground, they produce ARs. At the same time, because the Loess Plateau region has poor soil conditions, the organic matter content of apple orchards is less than 1%. Plants develop ARs to sustain their growth and development in order to achieve a high yield, which has a direct impact on crop yield. ARs arise from non-root organs, such as leaves and stems. AR formation is a complicated biological process influenced by several internal and external factors [1]. On an anatomical basis, the AR formation process starts with the induction stage, which necessitates distinct cell divisions; at the same time, it involves the reprogramming of target cells to create new meristematic cells. Then, the induction stage is followed by the formation of AR primordia (initiation stage), and the final stage begins with primordia differentiation into a root, with differentiated vascular bundles connected to the stem’s vascular cylinder, and finishes with root emergence (AR emergence stage) [2,3]. Therefore, the formation process is divided into three stages: induction, initiation, and emergence. The induction stage is important for molecular reprogramming, and the initiation stage is vital for root primordia formation [4].

Plant roots play a vital role in the uptake of water and nutrients in the soil and respond to various stress signals [5]. Thus, root morphology is the fundamental feature that determines how plants acquire resources. To increase soil nutrient availability, roots need to expand their length and surface area for active nutrient absorption [6]. In agriculture, drought is a severe environmental stress, threatening plant development. In vitro, the tissue culture technique is valuable for analyzing tolerance or adapting to various stresses, which cannot be easily achieved in the field. Polyethylene glycol (PEG) is used to induce water stress in the medium [7]. Osmotic stress causes physiological malfunction by an abrupt shift in the solute concentration around the cell, triggering a sharp change in water movement across its cell membrane, and it affects morphological characteristics, such as root length, root surface area, root volume, and root–shoot ratio [8,9]. Various studies have shown that root growth is severely hampered when the plants are exposed to water stress. Water stress decreased the root development and size of the root apical meristem (RAM), stimulating premature cell differentiation without changing the morphology of the stem cell niche [10]. 

Endogenous hormones, in particular, are highly involved in AR formation because they respond to changing environmental settings and establish a signaling network within the plant [11]. Exogenous auxin treatment is used to promote ARs while cytokinin (CTK) inhibits them [3,12]. Cytokinin–auxin crosstalk is also critical for controlling root meristem size, with auxin and CTK acting antagonistically in the formation of roots [13], which was mediated by SHORT HYPOCOTYL 2 (SHY2) [14]. Arabidopsis response regulator 1 (ARR1) directly binds to the SHY2 promoter region and stimulates CTK signaling. SHY2 degradation is activated in response to auxin to assist auxin transport and dispersion. Signaling components, such as histidine kinases (AHKs) and ARRs, are also engaged in the restrictive response of CTK during rooting [15,16]. Moreover, abscisic acid (ABA) mediates numerous critical developmental processes in plants. It has been shown to promote tolerance to various abiotic stresses, including drought, salt, and low temperature. Increased endogenous ABA content has been reported to negatively regulate ARs and LRs in apples and other crops [17,18,19,20]. Furthermore, the accumulation of jasmonic acid (JA) at the stem basal parts of petunia cuttings hampers AR formation [21]. However, the role of plant hormones, their homeostasis, and associated signaling pathways in regulating AR formation in apples has not been well understood until now.

A variety of studies on the physiological and molecular aspects of root formation under drought stress in other crops have been conducted, but the characterization of the underlying biochemical and cellular mechanisms responsible for the regulation of AR formation under water scarcity in apples is still lacking. Therefore, this study aims to identify the effect of PEG on the underlying mechanisms: how do endogenous hormones and related genes respond to PEG during the formation of apple ARs? To determine them, CK and PEG-treated cuttings were sampled at different time points to measure hormones and conduct transcriptome analysis. At present, this is the first study that reveals multiple genes and complex hormonal-mediated interactions with PEG during AR formation in apple cuttings.

## 2. Results

### 2.1. Morphological and Anatomical Changes by PEG Treatment during AR Formation

**Figure 1:**
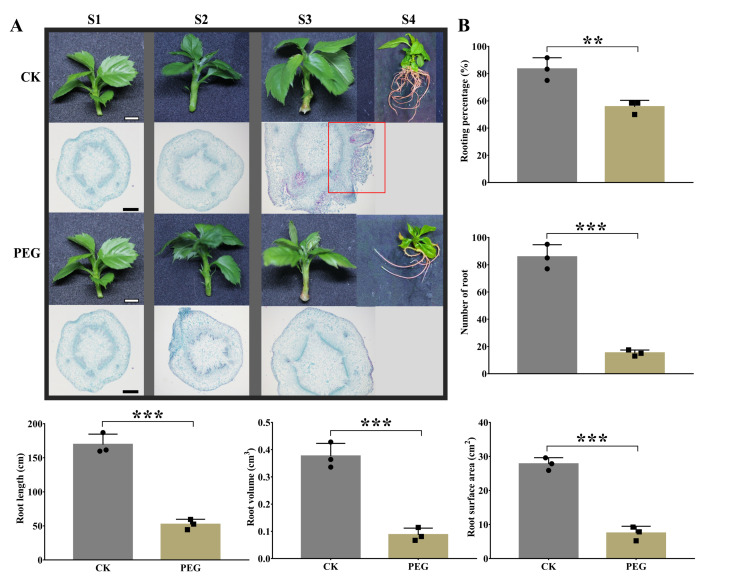
Morphological and anatomical observations were obtained at S1, S2, S3, and S4 after polyethylene glycol (PEG) treatment during adventitious root (AR) formation in GL-3 apple cuttings when compared to a PEG-free control (CK) group. The scale bar for morphology and microscopy photos was 1 cm and 500 μm, respectively (**A**). Effect of PEG on ARs’ development. Morphological parameters were measured at S4, including rooting percentage, number of roots, root length (cm), root volume (cm^3^), and root surface area (cm^2^) (**B**). Error bars refer to the average value ± SD from three biological replicates. Different asterisks indicate significant differences at ** *p* < 0.01 and *** *p* < 0.001.

A systemic analysis was performed on the formation of ARs in GL-3 apple cuttings using PEG at different time points to better understand the phenotypical changes in response to drought stress. Within S2, no morphological changes were observed in either group; however, at S3, the AR emergence stage was visible in CK cuttings but not in PEG-treated cuttings (Figure 1A). Moreover, a series of stem anatomy analyses were conducted to elucidate the cellular mechanisms underlying these morphological changes. Cross-sections of the samples indicated the formation of component cells at S1. On S2, mitosis was seen in cambial cells, and AR primordia were observed on S3 in the CK group, which was missing in PEG-treated cuttings (Figure 1A). Based on these results, AR formation can be divided into three stages, such as the induction stage, the initiation stage, and the emergence stage. 

To investigate how much PEG hampers AR development, we allowed stem cuttings to survive for 30 d (S4) on CK and PEG-containing medium. Subsequently, several AR phenotypical parameters were measured. The rooting percentage of PEG-treated cuttings was 55.55%, which was significantly lower than the CK cuttings. The number of ARs in the PEG group was only 15, which was 80.3% lower than CK cuttings (Figure 1B). The maximum root length of 169.4 cm in CK cuttings and 52.2 cm in PEG-treated cuttings was measured. Furthermore, root volume and surface area showed similar results with root length and root numbers, indicating that PEG treatment limits the development of ARs in GL-3 apple clones (Figure 1B).

### 2.2. RNA-Seq and Analysis of Differentially Expressed Genes

**Figure 2:**
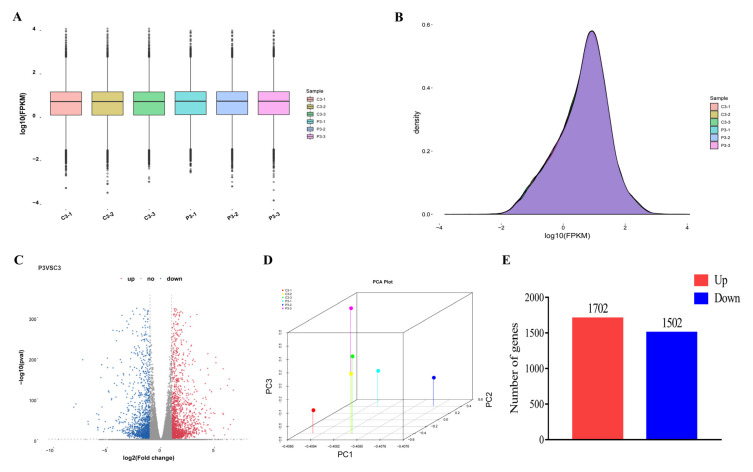
Expression levels of each sample. The boxplot showed the gene expression (**A**). The height of the curves represents density (**B**). The volcano indicates the differentially expressed genes (DEGs). Each dot in the figure signifies a particular DEG. The red dot shows upregulated DEGs. The blue dot indicates downregulated DEGs, and the dark grey dot is a nonsignificant differential gene (**C**). Principal Component Analysis (PCA) denotes gene expression levels (**D**). The number of upregulated and downregulated genes (**E**).

To characterize DEGs responsible for different root phenotypes (Figure 1) under CK and PEG treatment, mRNA analysis was executed on the stem basal parts of GL-3 at S2 (3 d) of treatment, which was critical for molecular reprogramming for ARs formation [22]. The statistical summary of three biological replications is shown in Tables S1 and S2. A total of 50.73–54.66 million reads were obtained. The percentages of Q20 and Q30 were more than 99.9% and 97.5%, respectively, and the GC content was 47% (Table S1). Furthermore, 92.84–93.22% mapped reads and 21.50–22.12% multiple mapped reads were found in all libraries, although 70.72–71.72% of the reads were uniquely mapped to the apple genome (Table S2). To investigate the various mechanisms underlying the CK and PEG responses, the R statistical package was used to assess gene expression levels and determine DEGs. All genes expressed by CK and PEG are shown in a boxplot and curve plots (Figure 2A,B). The distribution graph of up and down comparisons is presented, i.e., CK vs. PEG (Figure 2C). Additionally, a principal component analysis (PCA) graph was made for both groups, and a difference was found between them (Figure 2D). A total of 3204 DEGs were identified between CK and PEG, with 1702 upregulated and 1502 downregulated genes (Figure 2E).

### 2.3. Identification and Functional Annotation of DEGs Associated with AR Formation

**Figure 3:**
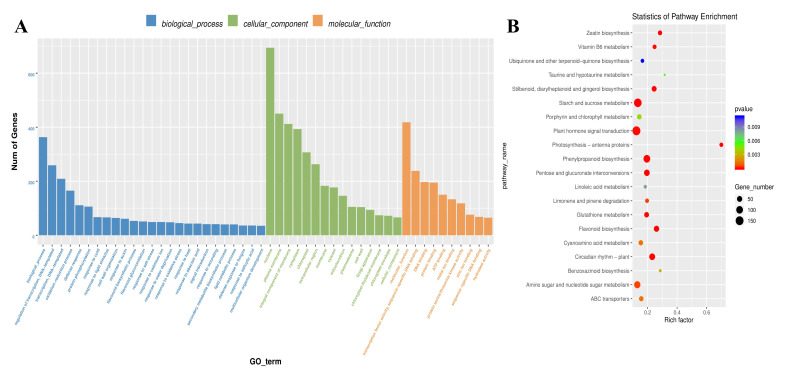
Gene classification was based on Gene Ontology (GO) analysis for differentially expressed genes (DEGs). Different classes are shown for biological processes, cellular components, and molecular functions (**A**). The Kyoto Encyclopedia of Genes and Genomes (KEGG) pathway of DEGs. The pathway names are provided on the vertical axis. The rich factor in the horizontal axis is the size of the point, which represents the number of DEGs, and the color of the dot represents the q value (**B**).

Gene Ontology (GO) and Kyoto Encyclopedia of Genes and Genomes (KEGG) analyses were performed to determine biological processes and functions enriched in DEGs. The DEGs were classified into three major GO categories: biological process, cellular component, and molecular function. A total of 50 terms (25 biological process, 15 cellular component, and 10 molecular function) were identified in the CK vs. PEG comparison (Figure 3A), illustrating the various DEGs’ roles in response to CK and PEG treatments. The primarily affected biological process category is biological process, regulation of transcription, and DNA-templated. Furthermore, nucleus, plasma membrane, and integral component of membrane were mainly affected in the cellular component category. In addition, molecular function, transcription factor activity, and sequence-specific DNA binding were primarily affected in the molecular function category (Figure 3A). Moreover, the KEGG pathway enrichment analysis of DEGs revealed that plant hormone signal transduction, starch and sucrose metabolism, and phenylpropanoid biosynthesis were the most enriched pathways (Figure 3B). Additionally, to analyze the cellular processes, we created a table of DEGs with pathway annotations related to hormone signaling and intracellular activities (Table 1). Following this, 1119 genes were engaged, with 524 (46.83%) and 595 (53.17%) being upregulated and downregulated, respectively, effectively by pathway, classifying them into hormone signal transduction, starch and sucrose metabolism, and amino sugar, nucleotide sugar metabolism, and others (Table 1).

### 2.4. Endogenous Hormone Measurement and Gene Expression of Hormone Signaling-Related Genes

The concentrations of endogenous hormones: IAA, ZR, ABA, JA-me, BR, and GA3 were measured at different stages of AR formation (S1, S2, and S3) in GL-3 apple cuttings after PEG treatment compared with the CK group. Furthermore, KEGG pathway analysis was used to identify hormone signaling-related genes that encode receptors and response factors. The detailed explanations are provided below. 

#### 2.4.1. Measurement of IAA and ZR and Analysis of Related DEGs

**Figure 4:**
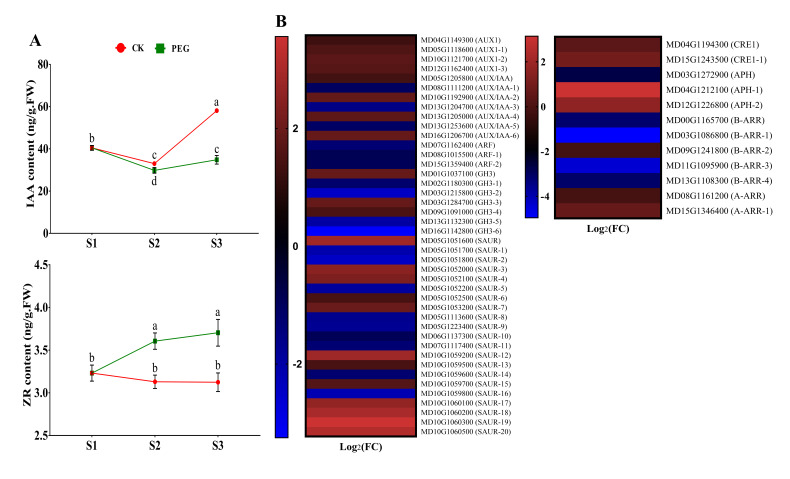
Effect of a polyethylene glycol (PEG) treatment on the endogenous content of indole-3-acetic acid (IAA) and zeatin riboside (ZR) over the course of this study (S1, S2, and S3) during adventitious root (AR) formation in GL-3 apple cuttings when compared to a PEG-free control (CK) group. Error bars refer to the average value ± SD from three biological replicates. Different letters indicate significant differences by least significant difference (LSD) test at *p* ≤ 0.05 (**A**). Selected differentially expressed genes (DEGs) related to auxin and cytokinin from RNA sequencing data (only at S2 of CK and PEG treatment). Heat map diagram of the log2FC, the red and blue colors specify up-and down-regulated expressions (**B**).

The physiological role of auxin is to stimulate the growth and development of plants, which requires auxin transport and signal transduction [23]. The endogenous content of IAA decreased from S1 to S2, and a sharp increase was observed at S3 in the CK group compared with PEG-treated cuttings (Figure 4A). CTK is antagonistic to auxin, where the concentration of ZR significantly increased from S1 to S3 in PEG-treated cuttings than in CK cuttings (Figure 4A). Moreover, four auxin influx carriers, *AUX1* (MD04G1149300, MD05G1118600, MD10G1121700, and MD12G1162400), were found in this study, and all were upregulated. Auxin responsive genes *AUX/IAA* were differentially expressed (up and down). Auxin response factors (*ARFs*): MD07G1162400, MD08G1015500, and MD15G1359400 were downregulated. Furthermore, *GH3* and *SAUR* members influence cell proliferation and plant growth downstream of *ARFs*, and some members were also downregulated (Figure 4B, Figure 7 and Table S3). Furthermore, PEG treatment activated CTK receptor *CRE*, including MD04G1194300 and MD15G1243500. The histidine-containing phosphotransfer *APH* encoding genes MD04G1212100 and MD12G1226800 were upregulated, a regulator for CTK signaling; however, MD03G1272900 was downregulated. In addition, type *A-ARR* genes (MD08G1161200 and MD15G1346400) were upregulated, while most *B-ARR* genes were downregulated (Figure 4B, Figure 7 and Table S4).

#### 2.4.2. Measurement of ABA and JA-me and Analysis of Related DEGs

**Figure 5:**
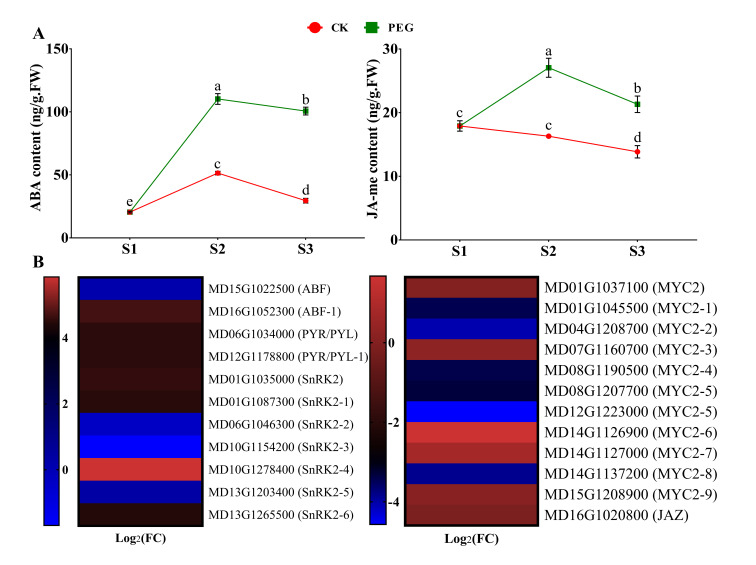
Effect of a polyethylene glycol (PEG) treatment on the endogenous content of abscisic acid (ABA) and methyl jasmonate (JA-me) over the course of this study (S1, S2, and S3) during adventitious root (AR) formation in GL-3 apple cuttings when compared to a PEG-free control (CK) group. Error bars refer to the average value ± SD from three biological replicates. Different letters indicate significant differences by least significant difference (LSD) test at *p* ≤ 0.05 (**A**). Selected differentially expressed genes (DEGs) related to ABA and JA from RNA sequencing data (only at S2 of CK and PEG treatment). Heat map diagram of the log2FC, the red and blue colors specify up-and down-regulated expressions (**B**).

ABA, a stress-responsive hormone, was found to be higher during all stages of AR formation. The same phenomenon appeared to occur for JA-me in response to PEG treatment compared with CK cuttings (Figure 5A). Additionally, ABA-related genes are mostly activated in response to abiotic stress conditions, mainly water deprivation, salt stress, and osmotic stress [24,25]. Abscisic acid receptor *PYR/PYL* MD06G1034000 and MD12G1178800 were both activated by PEG treatment. Most members of the ABA response factors, *ABFs* and *SnRK2s*, were also upregulated (Figure 5B, Figure 7 and Table S5). Moreover, jasmonate ZIM domain *JAZ* (MD16G1020800) was upregulated; however, the *MYC2* members were differentially regulated (up and down) (Figure 5B, Figure 7, and Table S6).

#### 2.4.3. Measurement of BR and GA and Analysis of Related DEGs

**Figure 6:**
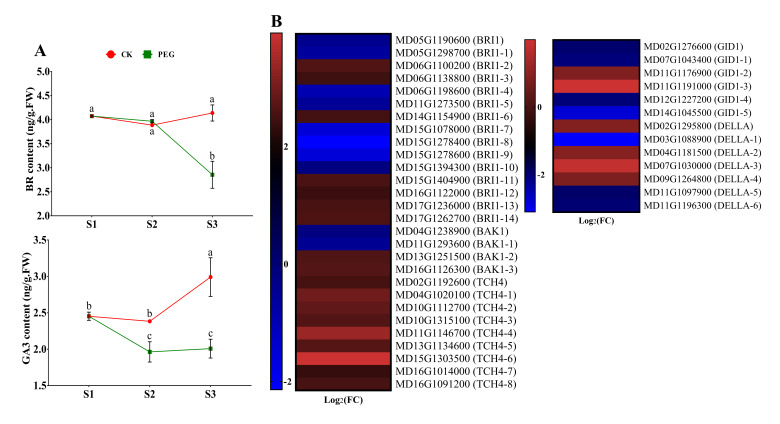
Effect of a polyethylene glycol (PEG) treatment on the endogenous content of brassinolide (BR) and gibberellic acid 3 (GA3) over the course of this study (S1, S2, and S3) during adventitious root (AR) formation in GL-3 apple cuttings when compared to a PEG-free control (CK) group. Error bars refer to the average value ± SD from three biological replicates. Different letters indicate significant differences by least significant difference (LSD) test at *p* ≤ 0.05 (**A**). Selected differentially expressed genes (DEGs) related to BR and GA from RNA sequencing data (only at S2 of CK and PEG treatment). Heat map diagram of the log2FC, the red and blue colors specify up-and down-regulated expressions (**B**).

**Figure 7:**
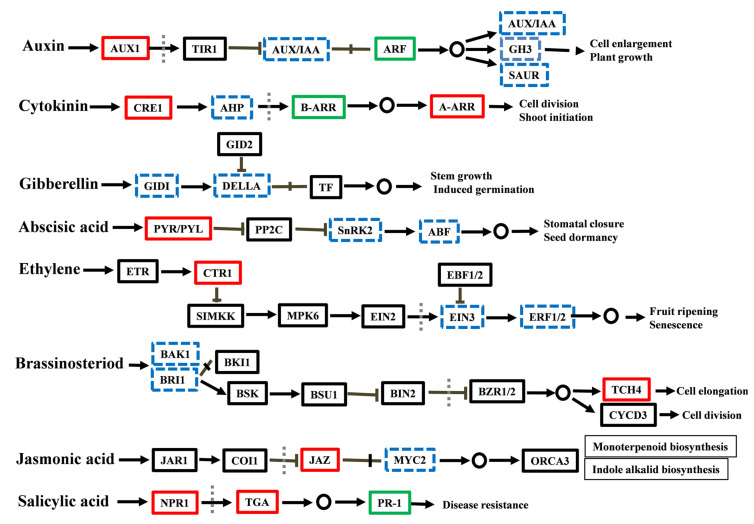
Differentially expressed genes (DEGs) were mapped to plant hormone signal transduction pathways in the KEGG database. The upregulated genes are indicated with red, downregulated with green, both up and downregulated with blue dashed boxes, and there are no DEGs with black line boxes. Vertical dashed lines depict the nucleus.

The existence of BR seems to have dual effects on plant development, both at concentration, where the contents have no significant difference at the early stages of AR formation in CK cuttings compared with PEG treated cuttings, but CK’s later stage was enriched and severe declined in PEG cuttings at S3 (Figure 6A). Furthermore, a difference was seen in GA3 concentration at S2, and the concentration was sharply increased at S3 in response to PEG-treated cuttings compared with the CK cuttings (Figure 6A). In addition, fifteen genes of brassinosteroid insensitive 1 were differentially regulated, of which eight were downregulated and seven were upregulated. Furthermore, *BAK1* MD04G1238900 and MD11G1293600 were downregulated, while on the other hand, MD13G1251500 and MD16G1126300 were upregulated. Interestingly, all *TCH* members were upregulated (Figure 6B, Figure 7 and Table S7). Moreover, gibberellin receptor *GID1* members were downregulated except for MD11G1176900 and MD11G1191000. However, most *DELLA* family members were upregulated except for three members (MD03G1088900, MD11G1097900, and MD11G1196300) (Figure 6B, Figure 7 and Table S8).

#### 2.4.4. Expression Analysis of ETH- and SA-Related DEGs

The expression levels of ETH and SA-related DEGs were also accessed from the RNA-seq data at S2 of CK and PEG treatment. ETH-related genes include *CTR1*, *EIN3*, *ERF1*, and *ERF2*, where the expressions of all *CTR1* genes were upregulated and *ERF1* genes were downregulated. Furthermore, the expressions of *EIN3* and *ERF2* were differentially regulated (up and down) (Figure S1, Figure 7 and Table S9). Only four SA-related genes were identified in this study, with *PR1* (MD05G1109100 and MD13G1265800) downregulated and *TGA* and *NPR1* (MD13G1053800 and MD17G1133200, respectively) upregulated (Figure S1, Figure 7 and Table S10).

### 2.5. Expression Analysis of Sugar Metabolism-Related DEGs

The concentration of sugar and the expression of metabolism-related genes were found to be associated with the energy status supply at the rooting stage. Because of this, KEGG pathway analysis was used to identify starch and sucrose metabolism-related genes, which contain the second-highest number of 138 genes after plant hormone signal transduction. Genes were selected and subjected to clustering analysis (Figure S2). According to Figure S2 and Table S11, 64.5% of genes were repressed by PEG treatment, including sucrose synthase *SUS* (MD02G1024600 and MD02G1109700), glucuronosyltransferase *GLT* (MD01G1143100, MD07G1209000, and MD07G1209300), starch synthase *STS* (MD10G1321800), callose synthase *CAS* (MD02G1319100 and MD07G1001800), and most members of cellulose synthase *CSA*, probable galacturonosyltransferase *PG*, pectinesterase *PCE*, and probable polygalacturonase *PP* (Figure S2 and Table S11). While some genes were activated by PEG treatment, they contain acid beta-fructofuranosidase *Ab-F* (MD06G1066600, MD07G1254500, and MD11G1195800), alpha, alpha-trehalase *AA-T* (MD01G1224000 and MD07G1294800), beta-glucosidase 47-like isoform X1 *B-G-47* and *18* (MD10G1316200 and MD11G1240900), and most members of the G-type lectin S-receptor-like serine *G-t L* (Figure S2 and Table S11). 

### 2.6. Expression Analysis of Root Development and Cell Cycle-Related Genes

Several genes involved in root growth and development were chosen to analyze their expression, as shown in Figure S3 and Table S12. Data revealed that 11 out of 19 genes were repressed by PEG treatment, mainly including glyceraldehyde-3-phosphate dehydrogenase *GAPCP1* (MD05G1234400 and MD10G1210900), nucleolin 1-like *N1-L* (MD16G1001600), nucleolin 2-like *N2-L* (MD13G1006400), phosphoethanolamine *N*-methyltransferase *PN-m* (MD06G1226700), and so on. Although some genes were expressed higher in response to PEG treatment, such as cryptochrome-1 isoform X1 *CIX1* (MD00G1201600), probable linoleate 9S-lipoxygenase 5 *PL9S* (MD16G1191300), and some more (Figure S3 and Table S12). Moreover, a cell is an organism’s core structural and functional unit. Thus, cell cycle-related genes were also studied. The data shows a similar trend with root development data, where most genes were repressed by PEG treatment, and some were activated. For example, cyclin-D1-1 *CYCD1* (MD16G1087700 and MD13G1087200), callose synthase 1 *CALS1* (MD07G1001800), callose synthase 3 *CALS3* (MD07G1002000), protein WALLS ARE THIN 1 *PWAT1* (MD15G1019700), and many more were repressed and patellin-3-like *Pate3L* (MD15G1406000), *Pate4L* (MD05G1265900), protein MEI2-like 1 isoform X1 *MEI2* (MD06G1131000), and some more genes were activated (Figure S3 and Table S13).

### 2.7. Validation of RNA-Seq Data Using RT-qPCR

**Figure 8:**
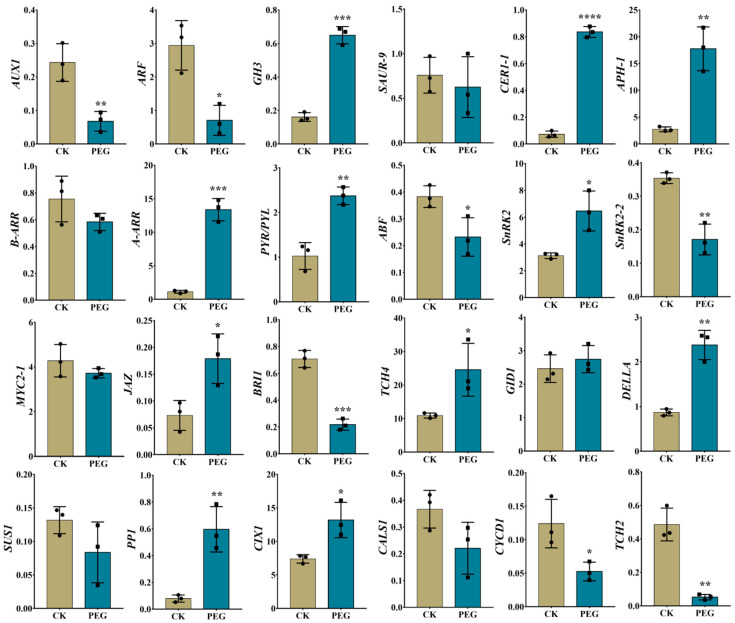
RT-qPCR-based results of randomly selected differentially expressed genes (DEGs) to validate the authenticity of the RNA sequencing data of polyethylene glycol (PEG) treated and PEG-free control (CK) treated cuttings at S2. Error bars refer to the average value ± SD from three biological replicates. Different asterisks indicate significant differences at ns *p* > 0.05, * *p* < 0.05, ** *p* < 0.01, *** *p* < 0.001, and **** *p* < 0.0001.

To validate the authenticity of the RNA-seq data, twenty-four DEGs related to auxin, CTK, ABA, JA, BR, GA, sugar, root development, and cell cycle were randomly picked for RT-qPCR examination. Notably, the relative expression patterns of all DEGs obtained from RT-qPCR were largely similar to those obtained from RNA-seq data (Figure 8), except for two DEGs (*AUX1* and *TCH2*) whose expressions were upregulated in RNA-seq data but RT-qPCR showed downregulated expressions. Therefore, the gene expression data obtained from RNA-seq is reliable.

## 3. Discussion

### 3.1. PEG Treatment Limits AR Formation in Apple GL-3 Cuttings

Water is essential for a plant’s survival and growth. Drought is a complex stress situation that severely limits a plant’s productivity and development based on the stress severity, duration, and developmental stage [26]. Indeed, water stress adaption has remained a central scientific concern in plant biology. The plants have developed sophisticated tolerance mechanisms that allow them to tolerate drought. PEG treatment inhibits root formation by reducing water potential [27]. In this work, AR primordia were missing at S3 in PEG-treated cuttings, and at S4, PEG produced fewer thin ARs than CK cuttings (Figure 1), which indicates that PEG detains ARs at initiation and emergence stages due to a decrease in cell enlargement and cell division. Compared with CK, PEG-treated cuttings significantly reduced the root length (Figure 1B), which might be related to the decline in turgidity and protoplasm dehydration, turgor loss, and limited cell expansion and division that function as a coping mechanism to survive water scarcity [28]. 

### 3.2. The Role of Hormonal Contents and Related DEGs in PEG-Induced AR Inhibition

The KEGG biological function pathways showed that numerous genes were involved in hormone signal transduction (Table 1). Hormonal and metabolic stimuli are the primary determinants of plant growth and development [29]. The auxin pathway is active in phases of embryonic and postembryonic root development in plants, extending from hypophysis to meristem initiation, emergence, and elongation, and includes polar auxin transport and auxin signal transduction mechanisms [30,31]. Auxin is believed to be important for the formation of ARs, and thus its high rate corresponds to high rooting. However, lower IAA content might be responsible for the low rate of AR formation in PEG-treated GL-3 cuttings (Figure 1 and Figure 4A). Previous studies revealed that auxin flow in plants is regulated by PIN1 and PIN2 localization. This is especially true of downward PIN1-dependent flow, which is related positively to the flow amount and growth rate [32,33]. Although PIN2, 4, and 7 have the ability to alter auxin distribution in the root elongation zone [33], the expression of these genes was not found in this study. Consistent with the results of previous studies, fewer ARs were found in PEG-treated cuttings. Auxin signal transduction stimulates downstream gene expression or interaction with other hormone signals upon auxin dispersion. In our study, most *AUX1* and *AUX/IAA* genes were upregulated, but, contrarily, *ARFs* were downregulated (Figure 4B, Figure 7 and Table S3). All the genes affected by auxin are controlled by the activation of *ARFs*. Therefore, downregulation of *ARFs* (MD07G1162400, MD08G1015500, and MD15G1359400) can be responsible for the low rate of ARs seen in the PEG-treated group (Figure 1). Antagonistic interactions between CTK and auxin are crucial for defining root meristem size. According to recent evidence, ZR is one of the primary inhibitors of roots [20]. In Arabidopsis, ZR restricts *PIN1* expression levels, regulating auxin polar transport during AR formation [34]. Polar transport of PIN protein sustains auxin content in the root tips, thereby controlling root growth and development [35]. According to these studies, the low concentration of IAA is mostly attributable to CK’s inhibitory effect in PEG-treated cutting (Figure 4A). CKs stimulate PIN1 degradation, particularly by interfering with its endocytic recycling in vacuoles and depleting its concentration on the plasma membrane [14]. In PEG-treated cuttings, AR formation was inhibited and was compatible with adaptations in CK signaling components, including the triggering of *CREs* and *A-ARRs* (Figure 4B, Figure 7 and Table S4). Collectively, under PEG treatment, IAA and CTK had a complicated underlying regulatory mechanism during AR inhibition, which needs more research.

ABA is a stress-responsive hormone that is required for plant dormancy and stress tolerance. Several studies have revealed that ABA has a negative effect on root formation and development [17,18]. Compared with CK cuttings, PEG-treated cuttings hold significantly higher ABA content at all time points during the formation of ARs (Figure 5A). Under abiotic stress, higher endogenous ABA contents have been observed in a variety of plant species under drought stress [36]. In a previous study, exogenous application of ABA reduced the inhibiting effect on roots caused by PEG, suggesting that ABA enhanced drought tolerance [37], which might be tied to its protective functions: stomatal closure, decreased evapotranspiration, and solute uptake [38]. Further analysis showed that ABA-related gene expression, which promotes dormancy and stress responses, was increased in PEG-treated cuttings (Figure 5B, Figure 7 and Table S5). Furthermore, JA has been identified as a suppressive factor for the formation of ARs in Arabidopsis that acts downstream of the auxin pathway [39]. Comparable findings were also reported in apples, where increased JA accumulation at the stem basal sections of the apple rootstock prevents AR formation [17,20]. The concentration of JA-me and the expression of related genes were considerably higher in PEG-treated cuttings, showing that increased JA-me accumulation is detrimental for AR formation in GL-3 apple cuttings (Figure 5A,B, Figure 7 and Table S6). In addition, the presence of BR had a twofold effect on root formation and development. A high concentration has an inhibitory effect, while a moderate concentration has a stimulatory effect [40]. Within S2, there was no significant difference found between the two groups. However, the BR content rose in CK cuttings at S3. At higher concentrations, GAs are also antagonistic to ARs [41]. The experiment revealed that exogenous GA application prevents ARs, and rice mutants lacking GA biosynthesis produce more ARs than wild-type [42]. However, in this study, we found opposing results: the concentration of GA3 was higher in CK cuttings, showing that PEG treatment inhibits the endogenous contents of GA3 (Figure 6A). Moreover, about half of the genes related to ETH and SA were upregulated by PEG treatment (Figure S1 and Tables S9 and S10). Taken together, the specific regulatory mechanisms regulating GA3, BR, ETH, and SA in the inhibition of ARs in apples remain unknown, which needs in-depth study.

### 3.3. PEG Inhibited ARs by Repressing Sugar Metabolism-Related DEGs

The KEGG biological function pathway study showed that most starch and sucrose metabolism-related genes were repressed by the PEG treatment (Table 1), indicating that molecular reprogramming and AR formation rate were restrained at both subcellular and transcriptional levels. Even so, more work is required for further biochemical verification. Sugar works as a major energy source and a vital signaling molecule that stimulates the plant’s developmental process [43]. In apple cuttings, the sugar content influenced the process of AR regeneration, and the sucrose content affected AR numbers [44]. The expression of 89 genes from 138 genes was repressed by PEG treatment, including *SUS1*, *SUS2*, *GLT1*, *GLT2*, *STS*, *CAS1*, and *CAS2* (Figure S2 and Table S11). Sucrose synthase (SUS) catalyzes a reversible conversion reaction that produces UDP-glucose and fructose, associated with sink strength determination and storage functions [45,46,47]. The expression of *SUS1* (MD02G1024600) and *SUS2* (MD02G1109700) was repressed by PEG treatment, which might be a result of the localization of low soluble sugar content in the stem basal parts during the induction stage of AR formation.

### 3.4. PEG Inhibited ARs by Repressing Root Development and Cell Cycle-Related Genes

Active cell division and differentiation occur mainly in the root meristem zone, which may influence the rate of root growth and development [23,48,49]. In terms of root formation, the expression of cell division and differentiation-related genes was repressed (Figure S3 and Table S12), which was thus compatible with the PEG-treated cuttings phenotype (Figure 1). However, the cell cycle and cell division are insufficient to initiate the process of root and bud formation [19,50]. The processes of cell division, differentiation, and elongation are essential for root initiation and emergence, despite being confined to pericycle cells. Cell cycle-related genes like *CYCD1* (MD16G1087700) and *CYCD1a* (MD13G1087200) that may contribute to cell division promotion in response to signals released during AR formation were thus inhibited by PEG treatment. This data suggests that cell division and differentiation were limited in PEG-treated cuttings; this decrease could be explained by turgidity and protoplasm dehydration, a loss of turgor, and restricted cell expansion and division. The repression of most root development and cell cycle-related genes might be a direct signal of PEG treatment inhibiting AR formation in GL-3 apple cuttings.

## 4. Materials and Methods

### 4.1. Plant Material and Treatment

The study was conducted at Northwest Agriculture and Forestry University, Yangling, China, and the stem cuttings of GL-3 apple [51] clones were selected as a specimen, obtained from Shenyang Agricultural University, China. Morphologically homogeneous GL-3 stem cuttings were cultured under sterile conditions on 1/2 strength MS medium under a 16/8 h light/dark cycle at 25 ± 1 °C, followed by 8 h at 15 ± 1 °C. The relative humidity was 70–80% [3,52]. In total, 630 GL-3 stem cuttings were cultured on ½ strength MS medium; sugar, 30 g/L; agar, 7.5 g/L; IBA, 1.2 mg/L; and pH 5.8; distributed into two groups. One group was treated with 6% PEG and referred to as PEG, and the second was served as control (CK). Samples from both groups were harvested at four different times, including 0 d, 3 d, 12 d, and 30 d, namely S1, S2, S3, and S4, respectively, indicating the different stages of AR formation: induction, initiation, and emergence stages [53]. According to our previous studies, 0.5 cm of stem basal tissue defines the rooting zone [54,55]. Approximately 90 stem cuttings were harvested at each time point from both groups (3 biological replications each containing 30 stem cuttings), immediately dipped in liquid nitrogen, and then stored at −80 °C for further analysis of hormones, RNA-sequencing, and RT-qPCR. 

### 4.2. Anatomical and Morphological Observations

Anatomical observations of the harvested stem basal parts at S1, S2, and S3 were made according to the earlier method [56]. Moreover, 36 stem cuttings (three biological replications each containing 12 stem cuttings) from each group were randomly selected for the measurement of root parameters at S4. For this, an Expression 10,000XL scanner (Epson, Sydney, Australia) was used to get AR scanned images; subsequently, WinRHIZO Pro root analysis software (WinRHIZO 2003, Quebec, Canada) was activated to analyze scanned images of ARs to measure root length (cm), root surface area (cm^2^), and root volume (cm^3^). The root numbers and rooting percentage (%) were measured manually [20,22]. 

### 4.3. Measurement of Endogenous Hormones

The endogenous abundances of six major hormones, IAA, JA-me, ABA, ZR, GA3, and brassinolide (BR), were measured at S1, S2, and S3 from both groups by the enzyme-linked immunosorbent assay (ELISA) method [57]. All monoclonal antibodies against each hormone were received from the Center of Plant Growth Regulator, China Agricultural University. For both groups, at each time point, three biological replications were made. A detailed description of the extraction and purification of hormones can be found in our previous study [58]. 

### 4.4. RNA Extraction, cDNA Synthesis, and Library Preparation

Samples were collected (three biological replications) for RNA sequencing at S2 after PEG treatment at LC-BIO TECHNOLOGIES (HANGZHOU) CO., LTD. Total RNA was extracted using Trizol reagent (Invitrogen, Carlsbad, CA, USA) following the manufacturer’s procedure. Total RNA quantity and purity were determined using a Bioanalyzer 2100 and an RNA 6000 Nano LabChip Kit (Agilent, Santa Clara, CA, USA) with a RIN number > 7.0. Approximately 10 ug of total RNA representing a specific adipose type was subjected to isolation of Poly (A) mRNA with poly-T oligo attached magnetic beads (Invitrogen). Following purification, the mRNA is fragmented into small pieces using divalent cations under elevated temperatures. Then the cleaved RNA fragments were reverse-transcribed to create the final cDNA library in accordance with the protocol for the mRNA Seq sample preparation kit (Illumina, San Diego, CA, USA). The average insert size for the paired-end libraries was 300 bp (±50 bp). And then, we performed the paired-end sequencing on an IlluminaHiseq4000 at the LC Sciences (Houston, TX, USA) following the vendor’s recommended protocol.

### 4.5. Identification of DEGs and Bioinformatics Analysis

The mapped reads of each sample were assembled by StringTie. Subsequently, all transcriptomes from samples were merged to reconstruct a comprehensive transcriptome using perl scripts. Following the completion of the transcriptome, the expression levels of all transcripts were estimated using StringTie and edgeR. StringTie was utilized to calculate the expression level of mRNAs using the FPKM. The differentially expressed mRNAs and genes were selected with log2 (fold change) >1 or log2 (fold change) <−1 and with statistical significance (*p*-value < 0.05) by the R package.

### 4.6. RT-qPCR Analysis

Expression analysis of several genes was conducted to confirm the RNA-seq data reliability. Three biological replications were examined. Our prior work describes the detailed process of RT-qPCR analysis [59,60,61]. The apple *actin* gene was used for normalization [4,62,63]. The relative expression was calculated by 2^ΔΔCt^ [64]. The pairs of primers are listed in Table S14.

### 4.7. Statistical Analysis

Figure 1B and Figure 8 were prepared and statistically analyzed by GraphPad Prism version 7.00. The results are shown as mean ± SD. ns *p* > 0.05, * *p* < 0.05, ** *p* < 0.01, *** *p* < 0.001, and **** *p* < 0.0001. The data in Figure 4A, Figure 5A and Figure 6A were analyzed by analysis of variance (ANOVA) and significant differences among the means were determined with the least significant difference (LSD) test at the 0.05% level by Statistics 8.1 software (Tallahassee FL 32317 USA), and GraphPad Prism prepared the figures.

## 5. Conclusions

This study elucidates the underlying mechanisms through which PEG treatment inhibits AR formation. To better understand the regulatory mechanisms, we measured hormone content and transcriptomic changes associated with developmental processes during AR formation. The specific biological functional analysis showed that AR formation under drought stress is a complex biological process involving the regulation of hormones and related DEGs, sugar metabolism, AR development, and cell cycle-related genes that ultimately control AR formation. Our findings lay the groundwork for further investigation into the roles of candidate genes in the multiple pathways that govern AR formation.

## Figures and Tables

**Table 1:** KEGG pathways are related to hormone signaling and intracellular activity.

Pathway Name	Pathway ID	Gene Number	Upregulated	Downregulated
Plant hormone signal transduction	ko04075	142	78	64
Starch and sucrose metabolism	ko00500	138	49	89
Amino sugar and nucleotide sugar metabolism	ko00520	84	23	61
Circadian rhythm-plant	ko04712	71	41	30
Carbon metabolism	ko01200	58	21	37
Flavonoid biosynthesis	ko00941	56	27	29
Biosynthesis of amino acids	ko01230	51	16	35
Glycerolipid metabolism	ko00561	40	30	10
Porphyrin and chlorophyll metabolism	ko00860	35	22	13
ABC transporters	ko02010	33	15	18
RNA transport	ko03013	30	14	16
Zeatin biosynthesis	ko00908	29	11	18
Glycolysis/Gluconeogenesis	ko00010	27	6	21
Fructose and mannose metabolism	ko00051	25	8	17
alpha-Linolenic acid metabolism	ko00592	24	7	17
Vitamin B6 metabolism	ko00750	23	12	11
Limonene and pinene degradation	ko00903	22	16	6
Photosynthesis-antenna proteins	ko00196	21	21	0
Pentose phosphate pathway	ko00030	20	6	14
Terpenoid backbone biosynthesis	ko00900	19	10	9
Carotenoid biosynthesis	ko00906	18	11	7
mRNA surveillance pathway	ko03015	17	9	8
beta-Alanine metabolism	ko00410	16	6	10
Phenylalanine metabolism	ko00360	15	5	10
Nitrogen metabolism	ko00910	14	7	7
Sulfur metabolism	ko00920	13	8	5
Cutin, suberine and wax biosynthesis	ko00073	12	8	4
Diterpenoid biosynthesis	ko00904	11	7	4
Photosynthesis	ko00195	10	9	1
Base excision repair	ko03410	9	6	3
Brassinosteroid biosynthesis	ko00905	8	3	5
Citrate cycle (TCA cycle)	ko00020	7	3	4
RNA polymerase	ko03020	6	1	5
Indole alkaloid biosynthesis	ko00901	5	2	3
Phagosome	ko04145	4	3	1
Flavone and flavonol biosynthesis	ko00944	3	2	1
Basal transcription factors	ko03022	2	0	2
Histidine metabolism	ko00340	1	1	0
Total		1119 (100%)	524 (46.83%)	595 (53.17%)

## Data Availability

Not applicable.

## Supplementary Materials

The following are available online at https://www.mdpi.com/article/10.3390/ijms23020976/s1.
