# Supplementary material for: Transcriptome Analysis Reveals Multiple Genes and Complex Hormonal-Mediated Interactions with PEG during Adventitious Root Formation in Apple
Source: Int J Mol Sci. 2022 Jan 17;23(2):976. doi: 10.3390/ijms23020976 (PMC8779459; doi:10.3390/ijms23020976)
Supplement: Supplementary file 1 [file ijms-23-00976-s001.zip › ijms-1548281-supplementary.pdf]

Table S1. Summary of the quality of sequencing data obtained in each sample.

Sample	Raw read	Raw base	Clean reads	Clean bases	Q20%	Q30%	GC content%
C3_1	51693542	7.75G	46200440	6.93G	99.98	97.67	47
C3_2	51237050	7.69G	45618258	6.84G	99.99	97.52	47
C3_3	53686716	8.05G	47407606	7.11G	99.99	97.58	47
P3_1	54661594	8.20G	47500518	7.13G	99.99	97.59	47
P3_2	50737654	7.61G	43019886	6.45G	99.99	97.56	47
P3_3	52844934	7.93G	45306530	6.80G	99.99	97.68	47

Table S2. Summary of total, multiple and uniquely mapped reads obtained in each sample.

Sample	Valid reads	Mapped reads	Unique Mapped reads	Multi Mapped reads
C3_1	46200440	42891815(92.84%)	32672003(70.72%)	10219812(22.12%)
C3_2	45618258	42423568(93.00%)	32370404(70.96%)	10053164(22.04%)
C3_3	47407606	44106266(93.04%)	33840244(71.38%)	10266022(21.65%)
P3_1	47500518	44279275(93.22%)	33985761(71.55%)	10293514(21.67%)
P3_2	43019886	40101057(93.22%)	30853917(71.72%)	9247140(21.50%)
P3_3	45306530	42311522(93.39%)	32437345(71.60%)	9874177(21.79%)

Table S3. Selected differentially expressed genes related to auxin.

**Auxin related**	**Annotation**	**Log2FC**
MD04G1149300 (AUX1)	auxin influx carrier (AUX1 LAX family)	1.05
MD05G1118600 (AUX1-1)	auxin influx carrier (AUX1 LAX family)	1.37
MD10G1121700 (AUX1-2)	auxin influx carrier (AUX1 LAX family)	1.58
MD12G1162400 (AUX1-3)	auxin influx carrier (AUX1 LAX family)	1.50
MD05G1205800 (AUX/IAA)	auxin-responsive protein IAA	1.14
MD08G1111200 (AUX/IAA-1)	auxin-responsive protein IAA	-1.21
MD10G1192900 (AUX/IAA-2)	auxin-responsive protein IAA	1.72
MD13G1204700 (AUX/IAA-3)	auxin-responsive protein IAA	-1.73
MD13G1205000 (AUX/IAA-4)	auxin-responsive protein IAA	1.60
MD13G1253600 (AUX/IAA-5)	auxin-responsive protein IAA	-1.28
MD16G1206700 (AUX/IAA-6)	auxin-responsive protein IAA	1.75
MD07G1162400 (ARF)	auxin response factor	-1.46
MD08G1015500 (ARF-1)	auxin response factor	-1.09
MD15G1359400 (ARF-2)	auxin response factor	-1.13
MD01G1037100 (GH3)	Auxin-responsive GH3 family protein,	1.75
MD02G1180300 (GH3-1)	Auxin-responsive GH3 family protein,	-1.39
MD03G1215800 (GH3-2)	Auxin-responsive GH3 family protein,	-2.50
MD03G1284700 (GH3-3)	Auxin-responsive GH3 family protein,	1.76
MD09G1091000 (GH3-4)	Auxin-responsive GH3 family protein,	1.28
MD13G1132300 (GH3-5)	Auxin-responsive GH3 family protein,	-2.08
MD16G1142800 (GH3-6)	Auxin-responsive GH3 family protein,	-3.25
MD05G1051600 (SAUR)	SAUR family protein	2.76
MD05G1051700 (SAUR-1)	SAUR family protein	-2.20
MD05G1051800 (SAUR-2)	SAUR family protein	-2.50
MD05G1052000 (SAUR-3)	SAUR family protein	2.39
MD05G1052100 (SAUR-4)	SAUR family protein	2.17
MD05G1052200 (SAUR-5)	SAUR family protein	-2.01
MD05G1052500 (SAUR-6)	SAUR family protein	1.25
MD05G1053200 (SAUR-7)	SAUR family protein	1.81
MD05G1113600 (SAUR-8)	SAUR family protein	-1.82
MD05G1223400 (SAUR-9)	SAUR family protein	-1.87
MD06G1137300 (SAUR-10)	SAUR family protein	-1.12
MD07G1117400 (SAUR-11)	SAUR family protein	-1.47
MD10G1059200 (SAUR-12)	SAUR family protein	2.74
MD10G1059500 (SAUR-13)	SAUR family protein	1.26
MD10G1059600 (SAUR-14)	SAUR family protein	-1.42
MD10G1059700 (SAUR-15)	SAUR family protein	1.43
MD10G1059800 (SAUR-16)	SAUR family protein	-2.29
MD10G1060100 (SAUR-17)	SAUR family protein	2.55
MD10G1060200 (SAUR-18)	SAUR family protein	2.92
MD10G1060300 (SAUR-19)	SAUR family protein	3.56
MD10G1060500 (SAUR-20)	SAUR family protein	3.09

Table S4. Selected differentially expressed genes related to cytokinin.

**Cytokinin related**	**Annotation**	**Log2FC**
MD04G1194300 (CRE1)	histidine kinase AHK2_3_4	1.39
MD15G1243500 (CRE1-1)	histidine kinase AHK2_3_4	1.75
MD03G1272900 (APH)	histidine-containing phosphotransfer peotein	-1.38
MD04G1212100 (APH-1)	histidine-containing phosphotransfer peotein	3.14
MD12G1226800 (APH-2)	histidine-containing phosphotransfer peotein	2.18
MD00G1165700 (B-ARR)	two-component response regulator ARR-B family	-2.12
MD03G1086800 (B-ARR-1)	two-component response regulator ARR-B family	-4.96
MD09G1241800 (B-ARR-2)	two-component response regulator ARR-B family	-1.01
MD11G1095900 (B-ARR-3)	two-component response regulator ARR-B family	-4.10
MD13G1108300 (B-ARR-4)	two-component response regulator ARR-B family	-2.06
MD08G1161200 (A-ARR)	two-component response regulator ARR-A family	1.05
MD15G1346400 (A-ARR-1)	two-component response regulator ARR-A family	1.55

Table S5. Selected differentially expressed genes related to abscisic acid.

**Abscisic acid related**	**Annotation**	**Log2FC**
MD15G1022500 (ABF)	ABA responsive element binding factor	-1.15
MD16G1052300 (ABF-1)	ABA responsive element binding factor	1.89
MD06G1034000 (PYR/PYL)	abscisic acid receptor PYR/PYL family	1.20
MD12G1178800 (PYR/PYL-1)	abscisic acid receptor PYR/PYL family	1.22
MD01G1035000 (SnRK2)	serine/threonine-protein kinase SRK2	1.43
MD01G1087300 (SnRK2-1)	serine/threonine-protein kinase SRK2	1.14
MD06G1046300 (SnRK2-2)	serine/threonine-protein kinase SRK2	-1.30
MD10G1154200 (SnRK2-3)	serine/threonine-protein kinase SRK2	-1.69
MD10G1278400 (SnRK2-4)	serine/threonine-protein kinase SRK2	5.84
MD13G1203400 (SnRK2-5)	serine/threonine-protein kinase SRK2	-1.09
MD13G1265500 (SnRK2-6)	serine/threonine-protein kinase SRK2	1.02

Table S6. Selected differentially expressed genes related to jasmonic acid.

**Jasmonic acid related**	**Annotation**	**Log2FC**

MD01G1037100 (MYC2)	transcription factor MYC2	1.06
MD01G1045500 (MYC2-1)	transcription factor MYC2	-1.44
MD04G1208700 (MYC2-2)	transcription factor MYC2	-3.13
MD07G1160700 (MYC2-3)	transcription factor MYC2	1.15
MD08G1190500 (MYC2-4)	transcription factor MYC2	-1.36
MD08G1207700 (MYC2-5)	transcription factor MYC2	-1.09
MD12G1223000 (MYC2-5)	transcription factor MYC2	-4.57
MD14G1126900 (MYC2-6)	transcription factor MYC2	1.67
MD14G1127000 (MYC2-7)	transcription factor MYC2	1.33
MD14G1137200 (MYC2-8)	transcription factor MYC2	-2.57
MD15G1208900 (MYC2-9)	transcription factor MYC2	1.10
MD16G1020800 (JAZ)	jasmonate ZIM domain-containing protein	1.01

Table S7. Selected differentially expressed genes related to brassinosteroid.

**Brassinosteroid related**	**Annotation**	**Log2FC**
MD05G1190600 (BRI1)	protein brassinosteroid insensitive 1	-1.20
MD05G1298700 (BRI1-1)	protein brassinosteroid insensitive 1	-1.32
MD06G1100200 (BRI1-2)	protein brassinosteroid insensitive 1	1.54
MD06G1138800 (BRI1-3)	protein brassinosteroid insensitive 1	1.19
MD06G1198600 (BRI1-4)	protein brassinosteroid insensitive 1	-1.48
MD11G1273500 (BRI1-5)	protein brassinosteroid insensitive 1	-1.27
MD14G1154900 (BRI1-6)	protein brassinosteroid insensitive 1	1.27
MD15G1078000 (BRI1-7)	protein brassinosteroid insensitive 1	-1.81
MD15G1278400 (BRI1-8)	protein brassinosteroid insensitive 1	-2.14
MD15G1278600 (BRI1-9)	protein brassinosteroid insensitive 1	-1.82
MD15G1394300 (BRI1-10)	protein brassinosteroid insensitive 1	-1.09
MD15G1404900 (BRI1-11)	protein brassinosteroid insensitive 1	1.41
MD16G1122000 (BRI1-12)	protein brassinosteroid insensitive 1	1.10
MD17G1236000 (BRI1-13)	protein brassinosteroid insensitive 1	1.39
MD17G1262700 (BRI1-14)	protein brassinosteroid insensitive 1	1.46
MD04G1238900 (BAK1)	brassinosteroid insensitive 1-associated receptor kinase 1	-1.09
MD11G1293600 (BAK1-1)	brassinosteroid insensitive 1-associated receptor kinase 1	-1.23
MD13G1251500 (BAK1-2)	brassinosteroid insensitive 1-associated receptor kinase 1	1.50
MD16G1126300 (BAK1-3)	brassinosteroid insensitive 1-associated receptor kinase 1	1.59
MD02G1192600 (TCH4)	xyloglucan:xyloglucosyl transferase TCH4	1.31

MD04G1020100 (TCH4-1)	xyloglucan:xyloglucosyl transferase TCH4	2.15
MD10G1112700 (TCH4-2)	xyloglucan:xyloglucosyl transferase TCH4	1.85
MD10G1315100 (TCH4-3)	xyloglucan:xyloglucosyl transferase TCH4	1.56
MD11G1146700 (TCH4-4)	xyloglucan:xyloglucosyl transferase TCH4	2.94
MD13G1134600 (TCH4-5)	xyloglucan:xyloglucosyl transferase TCH4	1.64
MD15G1303500 (TCH4-6)	xyloglucan:xyloglucosyl transferase TCH4	3.93
MD16G1014000 (TCH4-7)	xyloglucan:xyloglucosyl transferase TCH4	1.00
MD16G1091200 (TCH4-8)	xyloglucan:xyloglucosyl transferase TCH4	1.35

Table S8. Selected differentially expressed genes related to gibberellin.

**Gibberellin related**	**Annotation**	**Log2FC**
MD02G1276600 (GID1)	gibberellin receptor GID1	-1.33
MD07G1043400 (GID1-1)	gibberellin receptor GID1	-1.50
MD11G1176900 (GID1-2)	gibberellin receptor GID1	1.23
MD11G1191000 (GID1-3)	gibberellin receptor GID1	1.99
MD12G1227200 (GID1-4)	gibberellin receptor GID1	-1.45
MD14G1045500 (GID1-5)	gibberellin receptor GID1	-2.59
MD02G1295800 (DELLA)	DELLA protein	1.26
MD03G1088900 (DELLA-1)	DELLA protein	-3.10
MD04G1181500 (DELLA-2)	DELLA protein	1.30
MD07G1030000 (DELLA-3)	DELLA protein	1.88
MD09G1264800 (DELLA-4)	DELLA protein	1.22
MD11G1097900 (DELLA-5)	DELLA protein	-1.27
MD11G1196300 (DELLA-6)	DELLA protein	-1.40

Table S9. Selected differentially expressed genes related to ethylene.

**Ethylene related**	**Annotation**	**Log2FC**
MD04G1066000 (CTR1)	serine/threonine-protein kinase CTR1	1.31
MD10G1130000 (CTR1-1)	serine/threonine-protein kinase CTR1	1.54
MD10G1130300 (CTR1-2)	serine/threonine-protein kinase CTR1	1.34
MD11G1033700 (CTR1-3)	serine/threonine-protein kinase CTR1	1.12
MD15G1006000 (CTR1-4)	serine/threonine-protein kinase CTR1	1.45
MD15G1146100 (CTR1-5)	serine/threonine-protein kinase CTR1	1.08
MD16G1197600 (CTR1-6)	serine/threonine-protein kinase CTR1	1.02

MD16G1050800 (EIN3)	ethylene-insensitive protein 3	3.10
MD16G1222100 (EIN3-1)	ethylene-insensitive protein 3	-2.43
MD04G1228800 (ERF1)	ethylene-responsive transcription factor 1	-1.08
MD05G1198700 (ERF1-1)	ethylene-responsive transcription factor 1	-1.03
MD10G1286200 (ERF1-2)	ethylene-responsive transcription factor 1	-1.20
MD13G1212900 (ERF1-3)	ethylene-responsive transcription factor 1	-3.35
MD13G1213100 (ERF1-4)	ethylene-responsive transcription factor 1	-2.73
MD16G1216900 (ERF1-5)	ethylene-responsive transcription factor 1	-2.04
MD04G1165400 (ERF2-6)	ethylene-responsive transcription factor 2	-1.39
MD05G1030800 (ERF2-7)	ethylene-responsive transcription factor 2	-1.16
MD07G1248700 (ERF2-8)	ethylene-responsive transcription factor 2	1.25
MD09G1252000 (ERF2-9)	ethylene-responsive transcription factor 2	-1.32
MD15G1334900 (ERF2-10)	ethylene-responsive transcription factor 2	1.03

Table S10. Selected differentially expressed genes related to salicylic acid.

**Salicylic acid related**	**Annotation**	**Log2FC**
MD05G1109100 (PR-1)	pathogenesis-related protein 1	-2.18
MD13G1265800 (PR-1-1)	pathogenesis-related protein 1	-2.71
MD13G1053800 (TGA)	transcription factor TGA	1.39
MD17G1133200 (NPR1)	regulatory protein NPR1	1.55

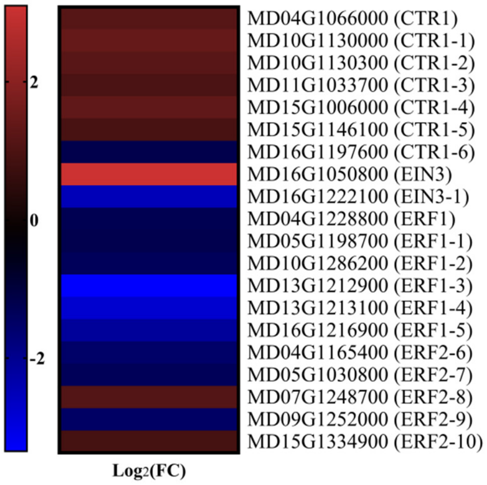

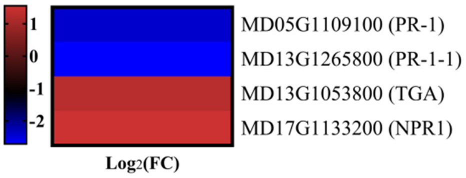

Figure S1. Selected DEGs related to ETH and SA from RNA sequencing data (only at S2 of CK and PEG treatment). Heat map diagram of the log2FC, the red and blue colors specify up-and down-regulated expressions.

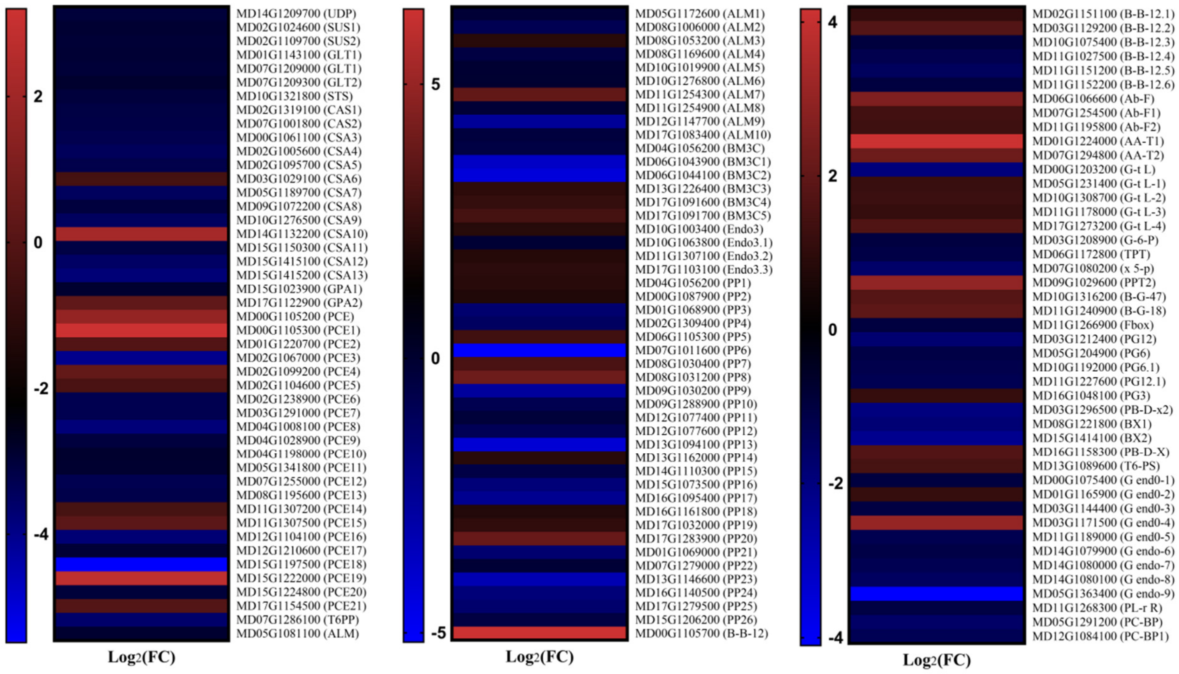

Figure S2. Selected DEGs related to starch and sucrose metabolism from RNA sequencing data (only at S2 of CK and PEG treatment). Heat map diagram of the log2FC, the red and blue colors specify up-and down-regulated expressions.

Table S11. Selected differentially expressed genes related sugar.

Sugar related	Annotation	Log2FC
MD14G1209700 (UDP)	UDPglucose 6-dehydrogenase	-1.27
MD02G1024600 (SUS1)	sucrose synthase	-1.11
MD02G1109700 (SUS2)	sucrose synthase	-1.18
MD01G1143100 (GLT1)	glucuronosyltransferase	-1.16
MD07G1209000 (GLT1)	glucuronosyltransferase	-1.24
MD07G1209300 (GLT2)	glucuronosyltransferase	-1.01
MD10G1321800 (STS)	starch synthase	-1.30
MD02G1319100 (CAS1)	callose synthase 1-like	-1.47
MD07G1001800 (CAS2)	callose synthase 1-like	-1.52

MD00G1061100 (CSA3)	cellulose synthase A catalytic subunit 4	-1.68
MD02G1005600 (CSA4)	cellulose synthase A catalytic subunit 4	-1.94
MD02G1095700 (CSA5)	cellulose synthase A catalytic subunit 4	-1.62
MD03G1029100 (CSA6)	cellulose synthase A catalytic subunit 4	1.07
MD05G1189700 (CSA7)	cellulose synthase A catalytic subunit 4	-2.02
MD09G1072200 (CSA8)	cellulose synthase A catalytic subunit 4	-1.34
MD10G1276500 (CSA9)	cellulose synthase A catalytic subunit 4	-2.06
MD14G1132200 (CSA10)	cellulose synthase A catalytic subunit 4	2.58
MD15G1150300 (CSA11)	cellulose synthase A catalytic subunit 4	-1.50
MD15G1415100 (CSA12)	cellulose synthase A catalytic subunit 4	-2.16
MD15G1415200 (CSA13)	cellulose synthase A catalytic subunit 4	-2.54
MD15G1023900 (GPA1)	glucose-1-phosphate adenylyltransferase	-1.05
MD17G1122900 (GPA2)	glucose-1-phosphate adenylyltransferase	1.49
MD00G1105200 (PCE)	pectinesterase	2.30
MD00G1105300 (PCE1)	pectinesterase	3.20
MD01G1220700 (PCE2)	pectinesterase	1.28
MD02G1067000 (PCE3)	pectinesterase	-3.05
MD02G1099200 (PCE4)	pectinesterase	1.56
MD02G1104600 (PCE5)	pectinesterase	1.15
MD02G1238900 (PCE6)	pectinesterase	-1.72
MD03G1291000 (PCE7)	pectinesterase	-1.72
MD04G1008100 (PCE8)	pectinesterase	-2.61
MD04G1028900 (PCE9)	pectinesterase	-1.37
MD04G1198000 (PCE10)	pectinesterase	-1.02
MD05G1341800 (PCE11)	pectinesterase	-1.05
MD07G1255000 (PCE12)	pectinesterase	-1.76
MD08G1195600 (PCE13)	pectinesterase	-1.61
MD11G1307200 (PCE14)	pectinesterase	1.09
MD11G1307500 (PCE15)	pectinesterase	1.41
MD12G1104100 (PCE16)	pectinesterase	-2.53
MD12G1210600 (PCE17)	pectinesterase	-1.20
MD15G1197500 (PCE18)	pectinesterase	-5.48
MD15G1222000 (PCE19)	pectinesterase	2.96
MD15G1224800 (PCE20)	pectinesterase	-1.28
MD17G1154500 (PCE21)	pectinesterase	1.29
MD07G1286100 (T6PP)	trehalose 6-phosphate phosphatase	-2.28
MD05G1081100 (ALM)	alpha-amylase	-1.04

MD05G1172600 (ALM1)	alpha-amylase	-1.12
MD08G1006000 (ALM2)	alpha-amylase	-1.68
MD08G1053200 (ALM3)	alpha-amylase	1.25
MD08G1169600 (ALM4)	alpha-amylase	-1.37
MD10G1019900 (ALM5)	alpha-amylase	-1.04
MD10G1276800 (ALM6)	alpha-amylase	-1.00
MD11G1254300 (ALM7)	alpha-amylase	3.05
MD11G1254900 (ALM8)	alpha-amylase	-1.10
MD12G1147700 (ALM9)	alpha-amylase	-3.12
MD17G1083400 (ALM10)	alpha-amylase	-1.28
MD04G1056200 (BM3C)	beta-amylase 3, chloroplastic	-1.40
MD06G1043900 (BM3C1)	beta-amylase 3, chloroplastic	-4.05
MD06G1044100 (BM3C2)	beta-amylase 3, chloroplastic	-4.45
MD13G1226400 (BM3C3)	beta-amylase 3, chloroplastic	1.38
MD17G1091600 (BM3C4)	beta-amylase 3, chloroplastic	1.60
MD17G1091700 (BM3C5)	beta-amylase 3, chloroplastic	2.16
MD10G1003400 (Endo3)	endoglucanase 3-like precursor	1.21
MD10G1063800 (Endo3.1)	endoglucanase 3-like precursor	-1.07
MD11G1307100 (Endo3.2)	endoglucanase 3-like precursor	1.13
MD17G1103100 (Endo3.3)	endoglucanase 3-like precursor	1.31
MD04G1056200 (PP1)	probable polygalacturonase	1.22
MD00G1087900 (PP2)	probable polygalacturonase	1.01
MD01G1068900 (PP3)	probable polygalacturonase	-2.25
MD02G1309400 (PP4)	probable polygalacturonase	-1.95
MD06G1105300 (PP5)	probable polygalacturonase	1.98
MD07G1011600 (PP6)	probable polygalacturonase	-5.17
MD08G1030400 (PP7)	probable polygalacturonase	2.29
MD08G1031200 (PP8)	probable polygalacturonase	3.40
MD09G1030200 (PP9)	probable polygalacturonase	-3.18
MD09G1288900 (PP10)	probable polygalacturonase	-1.63
MD12G1077400 (PP11)	probable polygalacturonase	-1.20
MD12G1077600 (PP12)	probable polygalacturonase	-1.78
MD13G1094100 (PP13)	probable polygalacturonase	-4.32
MD13G1162000 (PP14)	probable polygalacturonase	1.22
MD14G1110300 (PP15)	probable polygalacturonase	-1.43
MD15G1073500 (PP16)	probable polygalacturonase	-2.45
MD16G1095400 (PP17)	probable polygalacturonase	-2.93

MD16G1161800 (PP18)	probable polygalacturonase	1.06
MD17G1032000 (PP19)	probable polygalacturonase	1.52
MD17G1283900 (PP20)	probable polygalacturonase	3.17
MD01G1069000 (PP21)	probable polygalacturonase	-2.24
MD07G1279000 (PP22)	probable polygalacturonase	-1.12
MD13G1146600 (PP23)	probable polygalacturonase	-3.62
MD16G1140500 (PP24)	probable polygalacturonase	-2.46
MD17G1279500 (PP25)	probable polygalacturonase	-2.20
MD15G1206200 (PP26)	probable polygalacturonase	-1.35
MD00G1105700 (B-B-12)	beta-glucosidase 12-like precursor	6.37
MD02G1151100 (B-B-12.1)	beta-glucosidase 12-like precursor	1.00
MD03G1129200 (B-B-12.2)	beta-glucosidase 12-like precursor	1.68
MD10G1075400 (B-B-12.3)	beta-glucosidase 12-like precursor	-1.01
MD11G1027500 (B-B-12.4)	beta-glucosidase 12-like precursor	-1.28
MD11G1151200 (B-B-12.5)	beta-glucosidase 12-like precursor	-1.50
MD11G1152200 (B-B-12.6)	beta-glucosidase 12-like precursor	-1.03
MD06G1066600 (Ab-F)	acid beta-fructofuranosidase	2.57
MD07G1254500 (Ab-F1)	acid beta-fructofuranosidase	1.33
MD11G1195800 (Ab-F2)	acid beta-fructofuranosidase	1.27
MD01G1224000 (AA-T1)	alpha,alpha-trehalase	4.17
MD07G1294800 (AA-T2)	alpha,alpha-trehalase	2.21
MD00G1203200 (G-t L)	G-type lectin S-receptor-like serine/threonine-protein kinase	-2.00
MD05G1231400 (G-t L-1)	G-type lectin S-receptor-like serine/threonine-protein kinase	1.12
MD10G1308700 (G-t L-2)	G-type lectin S-receptor-like serine/threonine-protein kinase	1.20
MD11G1178000 (G-t L-3)	G-type lectin S-receptor-like serine/threonine-protein kinase	1.06
MD17G1273200 (G-t L-4)	G-type lectin S-receptor-like serine/threonine-protein kinase	1.64
MD03G1208900 (G-6-P)	glucose-6-phosphate/phosphate translocator 1, chloroplastic-like	-1.03
MD06G1172800 (TPT)	triose phosphate/phosphate translocator	-1.15
MD07G1080200 (x 5-p)	xylulose 5-phosphate/phosphate translocator	-1.70
MD09G1029600 (PPT2)	phosphoenolpyruvate/phosphate translocator 2	2.93
MD10G1316200 (B-G-47)	beta-glucosidase 47-like isoform X1	1.71
MD11G1240900 (B-G-18)	beta-glucosidase 18-like isoform X1	1.88
MD11G1266900 (Fbox)	F-box/LRR-repeat protein At3g58900-like isoform X1	-1.03
MD03G1212400 (PG12)	probable galacturonosyltransferase 12	-1.82
MD05G1204900 (PG6)	probable galacturonosyltransferase 6 isoform X1	-1.15
MD10G1192000 (PG6.1)	probable galacturonosyltransferase 6 isoform X1	-1.24
MD11G1227600 (PG12.1)	probable galacturonosyltransferase 12	-1.35

MD16G1048100 (PG3)	probable galacturonosyltransferase 3	1.06
MD03G1296500 (PB-D-x2)	probable beta-D-xylosidase 2	-2.03
MD08G1221800 (BX1)	beta-xylosidase/alpha-L-arabinofuranosidase	-1.88
MD15G1414100 (BX2)	beta-xylosidase/alpha-L-arabinofuranosidase	-2.30
MD16G1158300 (PB-D-X)	putative beta-D-xylosidase precursor	1.65
MD13G1089600 (T6-PS)	trehalose 6-phosphate synthase/phosphatase	1.44
MD00G1075400 (G end0-1)	glucan endo-1,3-beta-glucosidase 14	-1.04
MD01G1165900 (G end0-2)	glucan endo-1,3-beta-glucosidase 14	1.08
MD03G1144400 (G end0-3)	glucan endo-1,3-beta-glucosidase 14-like isoform X1	-1.07
MD03G1171500 (G end0-4)	glucan endo-1,3-beta-glucosidase-like	3.03
MD11G1189000 (G end0-5)	glucan endo-1,3-beta-glucosidase-like	-1.29
MD14G1079900 (G endo-6)	glucan endo-1,3-beta-glucosidase, basic isoform-like	-1.14
MD14G1080000 (G endo-7)	glucan endo-1,3-beta-glucosidase, basic isoform-like	-1.35
MD14G1080100 (G endo-8)	glucan endo-1,3-beta-glucosidase, basic isoform-like	-1.55
MD05G1363400 (G endo-9)	glucan endo-1,3-beta-glucosidase 5-like	-4.10
MD11G1268300 (PL-r R)	probable leucine-rich repeat receptor-like	-1.10
MD05G1291200 (PC-BP)	PLASMODESMATA CALLOSE-BINDING PROTEIN 1-like	-1.67
MD12G1084100 (PC-BP1)	PLASMODESMATA CALLOSE-BINDING PROTEIN 1-like	-1.36

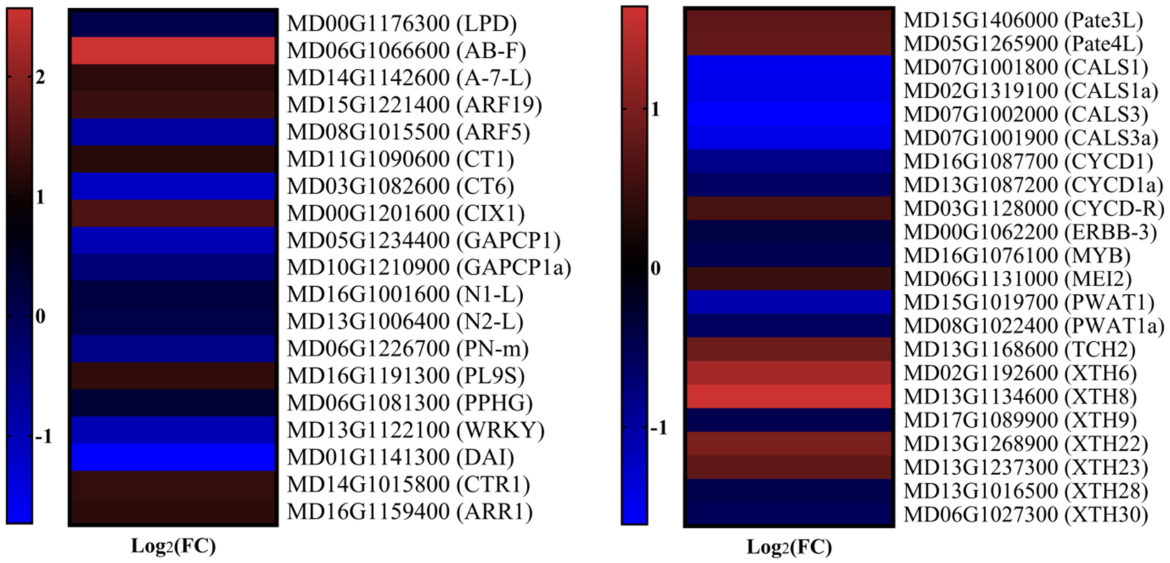

Figure S3. Selected genes related to root development and cell cycle from RNA sequencing data (only at S2 of CK and PEG treatment). Heat map diagram of the log2FC, the red and blue colors specify up-and down-regulated expressions.

Table S12. Selected differentially expressed genes related to root development.

Root development	Annotation	log2(fc)
MD00G1176300 (LPD)	14-3-3-like protein D	-0.51
MD06G1066600 (AB-F)	acid beta-fructofuranosidase	2.57
MD14G1142600 (A-7-L)	actin-7-like	0.53
MD15G1221400 (ARF19)	auxin response factor 19-like isoform X2	0.73
MD08G1015500 (ARF5)	auxin response factor 5-like	-1.09
MD11G1090600 (CT1)	copper transporter 1-like	0.51
MD03G1082600 (CT6)	copper transporter 6-like	-1.33
MD00G1201600 (CIX1)	cryptochrome-1 isoform X1	0.94
MD05G1234400 (GAPCP1)	glyceraldehyde-3-phosphate dehydrogenase GAPCP1	-1.21
MD10G1210900 (GAPCP1a)	glyceraldehyde-3-phosphate dehydrogenase GAPCP1	-0.82
MD16G1001600 (N1-L)	nucleolin 1-like	-0.44
MD13G1006400 (N2-L)	nucleolin 2-like	-0.48
MD06G1226700 (PN-m)	phosphoethanolamine N-methyltransferase 1-like	-0.92
MD16G1191300 (PL9S)	probable linoleate 9S-lipoxygenase 5	0.61
MD06G1081300 (PPHG)	probable phospholipid hydroperoxide glutathione peroxidase	-0.37
MD13G1122100 (WRKY)	probable WRKY transcription factor 75	-1.23
MD01G1141300 (DAI)	protein DA1-related 2 isoform X1	-1.73
MD14G1015800 (CTR1)	serine/threonine-protein kinase CTR1-like isoform X2	0.63
MD16G1159400 (ARR1)	two-component response regulator ARR1-like isoform X2	0.55

Table S13. Selected differentially expressed genes related to cell cycle.

Cell cycle	Annotation	log2(fc)
MD15G1406000 (Pate3L)	patellin-3-like	0.74
MD05G1265900 (Pate4L)	patellin-4-like isoform X2	0.79
MD07G1001800 (CALS1)	callose synthase 1	-1.52
MD02G1319100 (CALS1a)	callose synthase 1-like	-1.47
MD07G1002000 (CALS3)	callose synthase 3-like	-1.61
MD07G1001900 (CALS3a)	callose synthase 3-like	-1.46
MD16G1087700 (CYCD1)	cyclin-D1-1	-0.88
MD13G1087200 (CYCD1a)	cyclin-D1-1-like	-0.63
MD03G1128000 (CYCD-R)	cyclin-dependent kinases regulatory subunit 1	0.57
MD00G1062200 (ERBB-3)	ERBB-3 BINDING PROTEIN 1 isoform X1	-0.43

MD16G1076100 (MYB)	myb-like protein Q isoform X2	-0.52
MD06G1131000 (MEI2)	protein MEI2-like 1 isoform X1	0.46
MD15G1019700 (PWAT1)	protein WALLS ARE THIN 1	-1.09
MD08G1022400 (PWAT1a)	protein WALLS ARE THIN 1-like	-0.61
MD13G1168600 (TCH2)	xyloglucan 6-xylosyltransferase 2-like	0.86
MD02G1192600 (XTH6)	xyloglucan endotransglucosylase/hydrolase protein 6	1.31
MD13G1134600 (XTH8)	xyloglucan endotransglucosylase/hydrolase protein 8	1.64
MD17G1089900 (XTH9)	xyloglucan endotransglucosylase/hydrolase protein 9-like	-0.51
MD13G1268900 (XTH22)	xyloglucan endotransglucosylase/hydrolase protein 22-like	0.98
MD13G1237300 (XTH23)	xyloglucan endotransglucosylase/hydrolase protein 23	0.76
MD13G1016500 (XTH28)	xyloglucan endotransglucosylase/hydrolase protein 28	-0.49
MD06G1027300 (XTH30)	xyloglucan endotransglucosylase/hydrolase protein 30	-0.56

Table S14. Pair of primers used in this study

Gene name	Gene ID	Primer
AUX1-F	MD04G1149300	CCAGGAGCCTATAAAAGGGAAGG
AUX1-R	MD04G1149300	AAAGCGATGAAGCGAGTGAGA
CRE1-1-F	MD15G1243500	CTTGGAGGTGGGGATGGATG
CRE1-1-R	MD15G1243500	TTTGGGTGAACTGTGGCCTA
APH-1-F	MD04G1212100	CGGTACATGGCAGGAGGTTT
APH-1-R	MD04G1212100	TCCTTGATTACTTGCCAGCCT
B-ARR-F	MD00G1165700	CGACGATCCCACTTGGCTTA
B-ARR-R	MD00G1165700	TTGGCGAAGCAAGTGCAAAG
A-ARR-F	MD08G1161200	CTTCGGCCTTACCTGCTCAA
A-ARR-R	MD08G1161200	TTGACGGTAGTGCCCTTCAC
PYR/PYL-F	MD06G1034000	TTATCTCAGGGCTACCGGCT
PYR/PYL-R	MD06G1034000	CGCCCCCGATGATACTGAAA
ABF-F	MD15G1022500	TCGTCTTCCTCTACTGCGGA
ABF-R	MD15G1022500	GATTCGCGGTTCGATTGCAT
SnRK2-F	MD01G1035000	AGGACAAATGGAGTGGCGAG
SnRK2-R	MD01G1035000	CGGATGCTTCAAGGACCTGT
SnRK2-2-F	MD06G1046300	TTTGGTCCTGTGGTGTGACG
SnRK2-2-R	MD06G1046300	GGTATGGTGTACTGGGCACT
MYC2-1-F	MD01G1045500	TCCTTGAGCAGTGTGGAGAG
MYC2-1-R	MD01G1045500	CGTCTCTTCTTGGAGCGAGT
JAZ-F	MD16G1020800	GGCGGAAGCATTTTCCTTGT

JAZ-R	MD16G1020800	TCTCAGCCCACCACAATCAC
GID1-F	MD02G1276600	CCGCCTCAAGTCCAGTTCAT
GID1-R	MD02G1276600	CCGTAGGTTTTCGTTGACGC
DELLA-F	MD02G1295800	GCTTGAGTTGGCTGGGTTTG
DELLA-R	MD02G1295800	TTGTACCCGTCATAGCCACG
BRI1-F	MD05G1190600	TCGAGCAAGATTTCGAGCCC
BRI1-R	MD05G1190600	GCTCTGACGCCATGTATCCT
TCH4-F	MD02G1192600	GGCCATCCAACTCGTTCTTG
TCH4-R	MD02G1192600	TTCATGCTCACACGCCCAAA
SUS1-F	MD02G1024600	AACAAAACCGCAAACGCCAT
SUS1-R	MD02G1024600	CCGTGGTTGTGGTTGTGTAAC
PP1-F	MD04G1056200	TGTATGTGATGCTCCCGCTC
PP1-R	MD04G1056200	TCCTTCTCCACCAATCCCCA
CIX1-F	MD00G1201600	TGGAGGCGGCTATCTCAAAC
CIX1-R	MD00G1201600	GCGGATAAAGGGTCGGAAGT
CALS1-F	MD07G1001800	AAGCAGATCGGGCACAACTT
CALS1-R	MD07G1001800	ACTTCAACAGCCTCCGTCTG
CYCD1-F	MD16G1087700	ACACTCTCAGTCGCTTGACG
CYCD1-R	MD16G1087700	AACAGCGAAGGAACCAGAGG
TCH2-F	MD13G1168600	CTTCAACGACATCCGCGAAC
TCH2-R	MD13G1168600	CTCGGCGTAGTTGTTGGACT
ARF-F	MD07G1162400	GTTCATTGACTGGAAGGGGCGC
ARF-R	MD07G1162400	TCATGATCTGATGGTTATGGTGGTGA
GH3-F	MD01G1037100	TGAACTTAGTTGTCCTCCCCA
GH3-R	MD01G1037100	TCACGTAACATGTCAAAAATGTCTC
SAUR-9-F	MD05G1223400	CGCAGAAACGAATCGTTCCGTGTG
SAUR-9-R	MD05G1223400	GTTCAGGAGATCGTGCAGGTCATG
